# A second generation HIV-IN-EGFP fluorescent viral system to analyze HIV-1 in the nuclear compartment of infected cells

**DOI:** 10.1186/1742-4690-10-S1-P31

**Published:** 2013-09-19

**Authors:** Ashwanth Christopher Francis, Cristina Di Primio, Valentina Quercioli, Annegret Boll, Daniele Arosio, Anna Cereseto

**Affiliations:** 1 Centre for Integrative Biology (CIBIO), University of Trento, Trento, Italy; 2 Istituto di Biofisica, Consiglio Nazionale delle Ricerche, Trento, Italy; 3 Laboratory of Neurobiology, Scuola Normale Superiore, Pisa, Italy

## Background

To study the nuclear biology of HIV-1 we recently developed a microscopy based fluorescent HIV-IN-EGFP system [1]. HIV-IN-EGFP exploits the Vpr mediated *trans*-incorporation to incorporate IN-EGFP in viral particles made with pNL-IN-D64E. The ability to visualize HIV-IN-EGFP within the nucleus makes it an attractive tool to study quantitatively the nuclear import step of HIV-1 [2,3]. In this work we provide new evidence for efficient visualization of HIV-1 complexes in the nuclei of infected cells through a new optimized system.

## Methods

In order to optimize HIV-IN-EGFP infectivity various modifications were introduced into the visualization system. The functional tetramerization of IN mutations E11K and K186E [4] was introduced into Vpr-IN-EGFP constructs and nuclear detection of functional tetramers of HIV-IN-EGFP was evaluated in Hela-P4 cells. In addition the EGFP was replaced in Vpr-IN with super-folder GFR. Infectivity of HIV-IN-EGFP was verified in 293T cells at 3 days post infection.

## Results

Since the HIV-IN-EGFP visualization system [1] is produced by IN-EGFP *trans*-incorporation through Vpr, we have better analyzed the impact of this manipulation on viral infectivity. We observed that Vpr-IN-EGFP molecules are able to efficiently complement D64E mutant integrase for infectivity, however the infectivity values of *trans*-incorporated viruses are low with respect to the NL4.3 virus. Through *trans*-complementation of NL4.3 virus we find that incorporation of IN alone or fused with EGFP has a lethal effect in native wild type virions by severely affecting their infectivity. Further, by modifying the tetramerization properties of IN-EGFP [4] we attempted to improve viral infectivity. In fact, as previously suggested by Hare *et al. *[4], trans-incorporation of integrase molecules mutated in the N-terminus (E11K) and catalytic domain (K186E) induces stabilization of the integrase tetramer leading to a more functional complex. Confocal microscopic analysis revealed that functional tetramerization of IN-EGFP results in a loss of GFP fluorescence that can be recovered through the use of brighter fluorophores such as the super-folder GFP. Finally, the improved infectivity of the *trans*-incorporated viral particles was complemented with a dual labeling, which allows monitoring the different monomers within the integrase complex.

## Conclusions

Until recently the nuclear biology of HIV-1 has been investigated through molecular techniques, with the development of HIV-IN-EGFP it is possible to assess the localization of HIV-1 in the nuclear compartment. This technique allowed the first determination that HIV-1 localizes in the de-condensed regions of the chromatin and near the nuclear rim [1]. In this work we exploited improved tetramerization properties of integrase [4] to develop a second-generation HIV-IN-EGFP, which preserves the same imaging properties as the first-generation even though the infectivity is significantly improved.
